# Comparison of Sperm Telomere Length between Two Sperm Selection Procedures: Density Gradient Centrifugation and Zeta Potential

**DOI:** 10.22074/ijfs.2020.5981

**Published:** 2020-02-25

**Authors:** Razieh Ghorbani-sini, Tayebeh Izadi, Marziyeh Tavalaee, Leila Azadi, Mehdi Hajian, Mahshid Rahimi Zamani, Mohammad Hossein Nasr-Esfahani

**Affiliations:** 1 ACECR Institute of Higher Education (Isfahan Branch), Isfahan, Iran; 2 Department of Reproductive Biotechnology, Reproductive Biomedicine Research Center, Royan Institute for Biotechnology, ACECR, Isfahan, Iran; 3 Department of Cellular Biotechnology, Cell Science Research Center, Royan Institute for Biotechnology, ACECR, Isfahan, Iran; 4 Department of Reproduction and Development, Royan Institute for Biotechnology, ACECR, Isfahan, Iran; 5 Isfahan Fertility and Infertility Center, Isfahan, Iran

**Keywords:** Density Gradient Centrifugation, DNA Fragmentation, Telomere

## Abstract

**Background:**

Telomeres are particular sequences of DNA located at the end of the eukaryotic chromosomes that are essential for genome integrity. Telomere length in spermatozoa differs among males, as well as spermatozoa. Also, decreased telomere length in spermatozoa of infertile men is associated with the reduction of fertility potential and embryo quality. Density gradient centrifugation (DGC) and swim-up are useful techniques for separation of spermatozoa with longer telomeres. Also, the selection of sperm based on surface negative electric charge or “Zeta potential”, can separate high percentage of spermatozoa with intact chromatin compared to DGC alone, and also the combination of DGC-Zeta can improve clinical outcomes of infertile men candidate for intracytoplasmic sperm injection (ICSI). Therefore, we compared sperm telomere length and DNA fragmentation between two sperm preparation procedures, namely DGC and zeta potential.

**Materials and Methods:**

In this experimental study, we assessed sperm telomere length and DNA fragmentation by quantitative real-time polymerase chain reaction (PCR) and TUNEL assay methods, respectively. The spermatozoa were obtained from infertile men with normozoospermia between September 2017 and December 2017 and prepared either by DGC or zeta potential methods. Sperm telomere length was expressed as relative and absolute units.

**Results:**

Compared with washed semen samples or control, no significant (P>0.05) difference was observed in the mean relative or absolute sperm telomere length when the two methods DGC or zeta potential were compared. However, the mean percentage of DNA fragmentation was significantly (P<0.05) lower in spermatozoa prepared by DGC or zeta potential methods than spermatozoa obtained from control samples.

**Conclusion:**

This is the first study that compared the effect of DGC and zeta potential as the sperm preparation methods on sperm telomere length. It seems that both methods can select sperm population with high DNA integrity and the same sperm telomeres length.

## Introduction

Lack of pregnancy following one year of unprotected sexual intercourse is termed” infertility”, and its frequency is around 15%, that 40% of which is related to male infertility factors. Male infertility can be cured by intracytoplasmic sperm injection (ICSI), which almost bypasses all-natural selection barriers that sperm faces during natural fertility (1).

Quality of oocyte and sperm are two critical parameters determining ICSI outcomes. Quality of sperm is commonly defined based on the assessment of routine seminal indices, such as sperm concentration, motility, and morphology which reflect the efficiency of the male reproductive system (2, 3). During ICSI, despite the selection of motile or viable spermatozoa with normal morphology, the overall outcome remains limited. This dearth partly contributes to other functional aspects of spermatozoa, especially the genomic integrity of these cells, as this structure approximately participates in 50% of the genetic constitutions of the next generation (4, 5). In this regard, Avendaño et al. (6) demonstrated that the spermatozoa with normal morphology may have DNA fragmentation. Therefore, sperm preparation or processing in addition to the selection of spermatozoa based on sperm functional characteristic may have significant effects on ICSI outcomes. When it comes to sperm selection, researchers have taken different approaches to choose the most "fecund or physiological" spermatozoa. This is one of the hot topics in the field of andrology. For further explorations, please refer to reviews published by Henkel (7), Rappa et al. (8), and Sakkas (9).

One of the approaches for the separation of functional spermatozoa according to cellular and molecular characteristics is the selection of sperm cells based on surface negative electric charge or “zeta potential”, which is induced by sialic acid added to sperm surface during maturation or the passage through the epididymis (10, 11). Selected sperm based on zeta potential has been shown to exhibit higher degrees of chromatin and DNA integrity compared to sperm selection based on the density gradient centrifugation (DGC) method and results in improved embryos quality (10, 12-14). In a randomized clinical study, it has been shown that the pregnancy rate was significantly higher when the combined methods of zeta potential and DGC procedures were applied in comparison with the DGC method alone in infertile men candidate for ICSI (15). Considering increased interest for clinical application of ICSI for severe male infertility, who are candidates for ICSI, there is urgent need to assess the molecular facets of sperm selection based on this technique compared to the DGC method.

Despite novel approaches for sperm selection/ preparation, routine sperm processing has a historical background and lies in the way of assisted reproductive techniques (ARTs), especially intrauterine insemination (IUI). Previous studies indicate that several approaches have been taken to process spermatozoa for insemination, including swim-up, swim-down, DGC, albumin gradient, glass wool filtration, and Sephadex beads (7- 9, 15). Among these techniques, the DGC method which separates spermatozoa based on their density (mass/ volume) exposed to the gradient in the centrifugation field is currently the most popular common technique in andrology (16-18). DGC is almost used for all types of ARTs including, IUI, *in vitro* fertilization (IVF), and ICSI due to several advantages, such as the clean fraction of highly mature and motile spermatozoa, and also, it can be used for processing of semen samples. Also, the DGC method removes leukocytes or other cells and markedly reduces reactive oxygen species (ROS) (17). However, one of the disadvantages of this technique is sperm exposure to shear forces during centrifugation which is believed to induce ROS, and it can lead to a decrease in genomic integrity of spermatozoa. However, this shortcoming could be partially resolved by supplementation of processing media with antioxidants when the DGC method is applied (19, 20).

One of the critical aspects of sperm selection/preparation procedures and sperm process techniques such as DGC is the genomic integrity of sperm cells. Spermatozoa have very highly condensed nucleus protected against any chemical and physical insults during *in vivo* or *in vitro* studies (7, 21). One of the cellular facets affecting genomic integrity is the telomere length.

Telomeres are guanine-rich sequences that are more prone to undergo DNA break than non-telomeric DNA regions. They are considered important targets for free oxygen radicals. In this line, several studies showed significant negative correlations between sperm telomere length and sperm parameters, such as DNA fragmentation, protamine deficiency, and oxidative stress (22-25). Besides, there are significant associations between sperm telomere length and the percentage of sperm motility and viability (25). Therefore, short telomere length in spermatozoa denotes different functional defects at the cellular and molecular levels. Several lines of evidence demonstrate significant positive correlations between sperm telomere length and other factors, such as male age, fertilization, and embryo quality (22, 25-27). Indeed, it has been shown that children born with short telomere length present a high load of genetic damages (28).

Considering the fundamental roles of the DGC method in andrology or ARTs, as well as Zeta potential for sperm preparation as a novel approach to select the most fecund sperm, we aimed, for the first time, to evaluate and compare the sperm telomere length as a parameter of sperm quality between DGC and Zeta potential methods used for sperm preparation.

## Materials and Methods

### Ethical approval and subjects

In this experimental study, was approved by the Research Ethics Committee of the Royan Institute (IR. ACECR.ROYAN.REC.1397.89). Between September 2017 and December 2017, semen samples were obtained from 15 infertile men with normozoospermia who referred to the Andrology Unit of the Isfahan Fertility and Infertility Center for semen analysis. Total sperm count, sperm concentration, sperm motility, and morphology of spermatozoa were equal to or above the lower reference limit according to the criteria for the selection of normozoospermia established by World Health Organization (WHO) (29). Men with leukocytospermia, age >40 years or other infertility-related diseases, such as varicocele, Y-chromosome microdeletion, a history of cryptorchidism and orchitis, abnormal hormonal profile, and semen samples with sperm autoantibodies were excluded from the study. Written informed consent was obtained from all participants.

### Sperm preparation

Semen samples were collected after 2-7 days of sexual abstinence and standard semen analysis was performed according to WHO (29). Each semen sample was aliquoted into three parts. The first part was considered “control” or “washed sample” group that was rinsed with VitaSperm (Inoclon, Iran). The second and third parts of the semen sample were processed by DGC and zeta potential methods, respectively. Then, sperm telomere length and DNA fragmentation were assessed by quantitative realtime polymerase chain reaction (PCR) and TUNEL assay, respectively.

### Sperm preparation by the density gradient centrifugation procedure

Semen samples were washed with sperm washing media (VitaSperm, Inoclon, Iran) supplemented with 10% human serum albumin. Then, the DGC procedure was performed with PureSperm (Nidacon International, Sweden). In this method, 1.5 ml of 45% PureSperm was layered over 1.5 ml of 90% PureSperm, and then, 1.5 ml washed samples were mounted on the 45% PureSpermand layer and centrifuged for 15 minutes (300 g). Subsequently, sperm pellet was regarded as processed spermatozoa and used for the assessment of sperm telomere length and DNA fragmentation (30).

### Sperm preparation by the zeta potential procedure

The zeta potential method was carried out based on a study conducted by Chan et al. (31). Briefly, semen specimens were rinsed with the serum-free VitaSperm processing medium, and their concentration was adjusted to 5×10^6^ spermatozoa/ml. Afterwards, 4 ml of adjusted sperm solutions were transferred to a 5-ml Falcon tube induced by gaining a positive surface charge using the rotation of the tube, two or three turns, inside a latex rubber tubing. One minute was specified for spermatozoa to adherence to the charged wall of the tube. Finally, the medium was collected to remove the non-adhering sperm cells.

Subsequently, the surface of the tube was washed thoroughly with VitaSperm plus 10% human serum albumin to detach adhering spermatozoa from the tube wall. Subsequently, the selected spermatozoa were centrifuged and used for further assessments.

### Evaluation of sperm DNA fragmentation using the TUNEL assay

For each sample, washed semen that obtained spermatozoa after DGC and zeta potential methods were used for assessment of DNA fragmentation according to the terminal deoxynucleotidyl transferase dUTP nick end labeling (TUNEL) assay (32). For conducting this method, a commercial detection kit was employed purchased from Promega company (Apoptosis Detection System Fluorescein, Promega, and Mannheim, Germany), and all the procedures were performed according to the manufacturer’s instructions. Lastly, the percentage of sperm DNA fragmentation for each group was evaluated under an Olympus fluorescent microscope (BX51, Japan). Spermatozoon without fragmented DNA or TUNELnegative spermatozoa were red, whereas spermatozoa with fragmented DNA or the TUNEL-positive were bright green.

### DNA extraction and telomere length measurement by quantitative real-time polymerase chain reaction

The extraction of DNA sperm and peripheral blood leukocytes were carried out by the QIAamp DNA Mini Kit (Qiagen, Italy) according to the manufacturer’s recommendations. Real-time PCR was performed according to the study by Cawthon (33). The results were expressed as the “relative telomere length” (2^-ΔΔct^) (33) and “absolute telomere length” according to a modified method introduced by O'Callaghan and Fenech (34).

### Statistical analyses

Statistical analyses were performed by the Statistical Program for Social Sciences (SPSS Inc., Version 11.0, Chicago, IL, USA). Data are expressed as the means and standard error of the mean (means ± SEM), except for the age reported as the standard deviation of the means (means ± SD). One-way ANOVA was used, followed by LSD t tests to analyze the differences of parameters before and after semen preparation. Pearson’s correlation coefficient was applied to calculate the association between different parameters. The P<0.05 was considered statistically significant.

For this study, the sample size was determined according to the sample size formula mentioned below:

$$n=\frac{{\left(Z_{1-\frac{\alpha }{2}}+Z_{1-\beta }\right)}^{2}*(\sigma_{1}^{2}+\sigma_{2}^{2})}{\left({µ}_{1}-{µ}_{2}\right)2}$$

In this formula, σ_1_=2.5; σ_2_=3.8; µ_1_=6.51; µ_2_=9.73, Z_1_- β=0.8, and ɑ=0.05. Accordingly, the minimum number of cases in each group was 15.

## Results

### Sperm characteristics and DNA fragmentation

**Fig 1:**
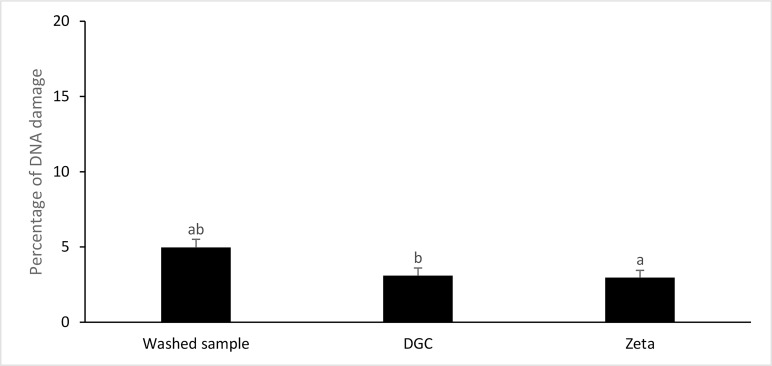
The comparison of the mean percentage of DNA fragmentation among washed samples, density gradient centrifugation (DGC), and zeta potential-processed samples. Common letter indicate significant differences between groups.

Table 1 shows the semen characteristics of 15 infertile men with normozoospermia that participated in this study. Sperm parameters, such as sperm concentration, motility, morphology, and semen volume, were higher than the defined threshold levels in accordance with the criteria established by the WHO (29). Sperm DNA fragmentation was assessed by the TUNEL assay, and the mean percentages of sperm DNA fragmentation were 4.97 ± 0.53, 3.10 ± 0.49, and 2.97 ± 0.47 in washed samples, DGC, and zeta potential groups, respectively. The analysis of the data revealed that the percentage of sperm DNA fragmentation was significantly lower in DGC and zeta potential-processed samples compared with the washed samples (P<0.05). Although, the percentage of sperm DNA fragmentation was lower in Zeta potential group compared with the DGC processed samples, but the difference was not statistically significant (Fig .1).

**Table 1:** Fresh semen characteristics of men with normozoospermia (n=15)


Parameters	Mean ± SE	Min	Max

Male age (Y)^*^	32 ± 5.02	25.00	45.00
Sperm concentration(10^6^/ml)	91.40 ± 4.1	70.00	125.00
Sperm count (10^6^/ejaculate)	339.34 ± 34.14	121.00	621.6
Sperm motility (%)	63.66 ± 1.5	55.00	70.00
Abnormal sperm morphology (%)	95.93 ± 0.43	92.00	97.00
Semen volume (ml)	3.78 ± 0.38	1.1	7.4


*; Mean ± SD.

### Sperm telomere length measurement

**Fig 2:**
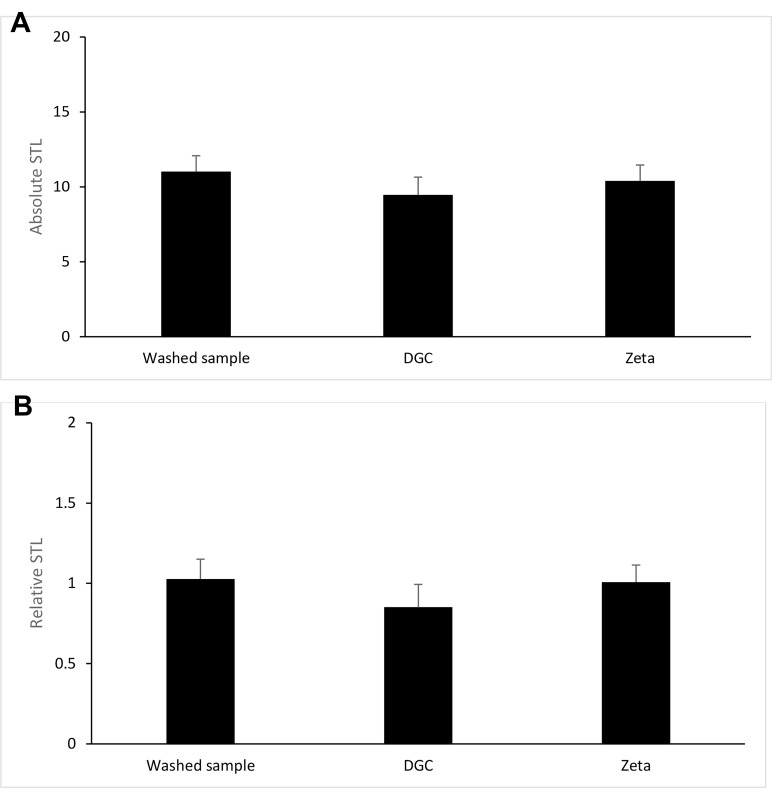
The comparison of sperm telomere length (STL) between experimental groups. A. Comparison of absolute and B. Relative of STL among washed semen samples, density gradient centrifugation (DGC), and zeta-processed samples (n=15).

The results of absolute and relative sperm telomere length among washed samples, DGC, and zeta potentialprocessed samples were compared (Fig .2). The mean absolute telomere length in the washed samples, DGC, and zeta potential-processed samples were 11.01 ± 1.06, 9.46 ± 1.18, and 10.39 ± 1.05, respectively. The differences among these groups were not statistically significant. Also, the mean relative telomere length in the washed samples, DGC, and zeta potential-processed samples were 1.02 ± 0.12, 0.85 ± 0.14, and 1.00 ± 0.1, respectively. The differences between the values of experimental groups were not statistically significant.

### Correlation between sperm telomere length and sperm parameters

The correlative analysis between absolute sperm telomere length and sperm parameters revealed a significant correlation between this parameter with sperm abnormal morphology (r=-0.561, P=0.03). The results of the correlation analysis of absolute and relative telomere length, sperm parameters, and sperm DNA fragmentation with the male age are presented in Table 2. The results indicated significant correlations of the male age with sperm abnormal morphology (r=-0.75, P=0.001), absolute (r=+0.64, P=0.009) and relative telomere length (r=+0.64, P=0.01).

**Table 2:** The correlation of male age with semen parameters, absolute, and relative sperm telomere length, as wel as sperm DNA fragmentation (n=15)


Parameters	r (P value)

Semen volume (ml)	0.17 (0.54)
Sperm concentration (×10^6^/ml)	0.31 (0.26)
Total sperm count (×10^6^)	0.29 (0.28)
Sperm motility (%)	-0.11 (0.69)
Abnormal sperm morphology (%)	-0.75 (0.001)
Sperm DNA fragmentation (%)	-0.008 (0.97)
Absolute sperm telomere length	0.64 (0.009)
Relative sperm telomere length	0.64 (0.01)


## Discussion

Numerous studies in the field of andrology emphasize on sperm telomere length as a sperm marker which has the ability to distinguish fecund sperm from non-fecund ones (22, 25, 35). In this regard, many studies have assessed the relationship between sperm telomere length and different sperm functional characteristics, showing that sperm telomere length has positive correlations with sperm count, sperm progressive motility, vitality, individual age, paternal, and the maternal age of the male parents at the time of conception and negative correlation with sperm DNA fragmentation and ROS production (22- 25). In this study, we also observed a significant negative correlation between absolute telomere length and the percentage of abnormal sperm morphology. Thus, this result has further emphasized on sperm telomere length as a positive marker for sperm quality. Unlike previous studies (36), we observed negative correlations between sperm telomere length with male age, indicating that similar to many sperm functional characteristics, this parameter is inversely associated with the male age.

As mentioned above, sperm selection/preparation procedures play a pivotal role in the management of ARTs and have profound effects on ICSI outcomes (7, 8). Previous studies have shown that the selection of sperm based on the surface electrical charge reduces the degree of sperm DNA fragmentation (10, 12). Therefore, we assessed the efficiency of zeta potential as a sperm selection procedure compared with DGC and neat semen in this study. As expected, and in accordance with the literature (10, 31), both techniques significantly reduced the degree of DNA fragmentation in the selected populations. Comparison of sperm DNA fragmentation in spermatozoa prepared by DGC and zeta potential methods showed a lower level of DNA fragmentation in spermatozoa prepared by the zeta potential technique, but such a difference was not statistically significant. This observation is in line with the previous literature (12, 31, 37) but a reduction (not statistically significant) may be due to population selection. In other studies, zeta potential and DGC procedures were conducted on semen samples obtained from infertile men with severe male fertility (12, 31, 37), while in this study, individuals were normozoospermic men according to WHO criteria due to minimizing heterogeneous factors (29).

Comparison of absolute and relative sperm telomere length among the three groups demonstrated the lack of a significant difference among experimental groups. In contrary to our results, Yang et al. (27) have shown that sperm processing by DGC and swim-up methods, presents higher telomere length. Although it is difficult to explain the differences between the two studies, one of the major differences in that study is the much higher population compared to our study. It is also important to note that in a study performed by Lafuente and colleagues (38), they used the fluorescent in-situ hybridization (FISH) technique to detect telomeres length. They failed to observe any difference among neat, DGC, and swimup a processed sample in normozoospermic individuals. They explain that the difference may be related to the methodology and sample size. However, another reason could be owing to the low oxidative stress levels, which account for shorter telomere length between experimental groups in different studies. In this study, due to the selection of normozoospermic individuals and the low mean of DNA fragmentation, it is not unexpected to observe any difference in telomere length between the groups.

In accordance with the literature, in this study, we detected a significant positive correlation between sperm telomere length and male age, indicating spermatozoa derived from old age men present higher telomeres length. It is also important to note that numerous factors, including oxidative stress, aging, psychological stress, obesity, infection, smoking, lifestyle, diet, etc., can affect telomere length (35, 38-40). Therefore, the contradiction observed in this study could be partially linked to these confounding factors and the low number of participants, considered one of the limitations of this study.

## Conclusion

The results of this study show that both DGC and zeta potential procedures can select sperm population with higher DNA integrity, but no difference was observed between the sperm selected samples in terms of telomeres length.
